# PPAR α and PPAR γ Polymorphisms as risk factors for Dyslipidemia in a Chinese han population

**DOI:** 10.1186/1476-511X-13-23

**Published:** 2014-01-26

**Authors:** Shu-Jun Gu, Zhi-Rong Guo, Zheng-Yuan Zhou, Xiao-Shu Hu, Ming Wu

**Affiliations:** 1 Center for Disease Control of Changshu, Suzhou, 215500, Jiangsu, China; 2 Department of Epidemiology, School of Public Health, Soochow University, Suzhou, Jiangsu 215123, China; 3 Health Bureau of Jiangsu Province, Nanjing, Jiangsu 21009, China; 4 Center for Disease Control of Jiangsu Province, Nanjing, Jiangsu 210009, China

**Keywords:** PPAR, Polymorphism, Dyslipidemia, Haplotype

## Abstract

**Background:**

The PPAR α and PPAR γ are the key messengers responsible for the translation of nutritional stimuli into changes for the expression of genes, particularly genes involved in lipid metabolism. However, the associations between PPAR α / γ polymorphisms and lipid serum levels in the general population were rarely studied, and the conclusions were conflicting. The objective was to investigate the associations of the PPAR α and PPAR γ polymorphisms with dyslipidemia.

**Methods:**

820 subjects were randomly selected from the Prevention of Multiple Metabolic Disorders and MS in Jiangsu Province cohort populations. The logistic regression model was used to examine the association between these polymorphisms and dyslipidemia. SNPstats was used to explore the haplotype association analyses.

**Results:**

In the codominant and log-additive models, rs1800206, rs1805192 and rs3856806 were all associated with dyslipidemia (P < 0.005). When the most common haplotype L-G (established by rs1800206, rs4253778) was treated as the reference group, the V-G haplotype was associated with dyslipidemia (P < 0.001), higher TC and TG levels (P < 0.01). Moreover, when compared to Pro-C haplotype (established by rs1805192, rs3856806), the Pro-T, Ala-C, Ala-T haplotypes were associated with dyslipidemia (p < 0.001). A-T haplotype was associated with higher TC levels, (p < 0.01), and the P-T, A-C, A-T haplotypes were associated with higher TG levels (p < 0.01).

**Conclusions:**

PPAR α and PPAR γ polymorphisms and haplotypes may be the genetic risk factors for dyslipidemia.

## Background

Cardiovascular disease (CVD)represents the leading cause of death in adults worldwide. Alterations in lipid and lipoprotein metabolisms have been investigated as important CVD risk factors [1]. It is well-known that high LDL-cholesterol (LDL-c) and triglycerides (TG) levels and low HDL-cholesterol (HDL-c) levels are strong predictable factors for cardiovascular events [2].Epidemiological data have indicated a continuous increase in prevalence rate of dyslipidemia during the last decades [1]. The exact cause of dyslipidemia is not known. Genetics play an important role on lipid homeostasis. The peroxisome proliferator-activated receptor (PPAR) isotypesare ligand-activated nuclear transcription factors, which have been identified that they play a critical physiological role as lipid sensors and regulators of lipid metabolism [3,4]. The PPAR α and PPAR γ were the key messengers responsible for the translation of nutritional stimuli into changes for the expression of genes, particularly genes involved in lipid metabolis [5]. PPAR α and PPAR γ are encoded by distinct genes and have specific tissue distribution.

PPAR α is mainly expressed in tissues with extensive fatty acid catabolism and its activation leads to changes in the transcription of multiple genes that regulate lipid and lipoprotein metabolism including LPL, APOC3, APOA1 and APOA5 [6,7]. Common variants of the PPAR α have been described. Rs1800206 has been associated with variation in lipid serum levels in Caucasian and Indian populations [8,9]. Another polymorphism, rs4253778 has also been found that it is associated with variation in lipid serum levels [10]. But Mazzotti et al. found that there were divergences on the association results regarding both polymorphisms between their two studied population (São Paulo City and Cuiaba City) [11,12].

PPAR γ is highly expressed in adipose tissue, where it plays an indispensable role in the regulation of adipocyte differentiation, lipid storage, glucose metabolism and the transcriptional regulation of a number of genes involved in these metabolic processes. Some of the key target genes of PPAR γ include the fat-specific ap2 gene, LPL, fatty acid transport, fatty acid binding protein, ABC-A1 [4,13]. The most studied of PPAR γ polymorphisms is rs1805192 [14]. Rs3856806 variant has been found to be associated with traits, which modulate the associations observed with the rs1805192 [15]. At present, many studies have reported that rs1805192 and rs3856806 polymorphisms were associated with obesity, insulin sensitivity and Type 2 diabetes [16-18]. However, the associations between rs1805192 or rs3856806 polymorphisms and lipid serum levels in the general population were rarely studied, and the conclusions were conflicting.

In the previous study, we have found that PPARα/γ polymorphisms (rs1800206, rs3856806 and rs1805192) were significantly associated with hypertriglyceridemia [19]. Thus, the purpose of this study was to further investigate the association of the four variants at the PPAR α (rs1800206, rs4253778 polymorphisms) and PPAR γ (rs3856806 and rs1805192 polymorphisms) locus with dyslipidemia. This study is the first to investigate the role of PPAR α and PPAR γ polymorphisms with dyslipidemia in Chinese Han population. Moreover, this is the first study that associated haplotypes of rs3856806 and rs1805192 polymorphisms with dyslipidemia.

## Results

Table 1 describes the characteristics of the studied population. Dyslipidemia participants exhibited a significantly higher mean age, BMI and WC than non-dyslipidemia population. The four SNPs were within Hardy–Weinberg equilibrium (P > 0.05). Linkage analysis showed there was no significant linkage disequilibrium between rs1800206 and rs4253778 or between rs1805192 and rs3856806 polymorphisms (D’ < 0.75).

**Table 1:** Baseline characteristics of the studied samples

**Variables**	**Non- dyslipidemia (N = 628)**	**Dyslipidemia (N = 192)**	**Total *(N = 820)***	**Statistical test values**	**p** ***- *** **Value**
Age (year)^a^	50.47 ± 9.51	48.65 ± 8.97	50.05 ± 9.40	2.354	0.019
Male^b^	204 (32.5)	66 (34.4)	270 (32.9)	0.238	0.626
Smoke N (%)^b^	160 (25.5)	45 (23.4)	199 (24.3)	0.326	0.568
Alcohol N (%)^b^	129 (20.5)	43 (22.4)	205 (25.0)	0.305	0.581
BMI (kg/m^2^)^a^	22.71 ± 3.14	23.77 ± 2.95	22.83 ± 3.12	-4.130	<0.001
WC (cm)^a^	76.44 ± 8.67	81.50 ± 9.21	77.00 ± 9.05	-6.978	<0.001

Note: means ± standard deviation for age, BMI, WC; BMI: body mass index; WC: waist circumference. ^a^Student’s t-test; ^b^chi-square test.

We further examined the potential associations between dyslipidemia and individual polymorphisms in PPAR α and PPAR γ based on two models (codominant and log-additive models; Table 2). In the codominant model, when the LL genotype of rs1800206 was used as the reference group, the LV genotype appeared to have a higher risk for dyslipidemia (the adjusted OR 5.75, 95% CI 3.91-8.46), whereas no association was observed to the VV genotype (adjusted OR 2.38; 95% CI 0.43-13.31). In the log-additive model, rs1800206 also appeared to be associated with dyslipidemia, the adjusted OR (95% CI) was 4.74(3.29-6.84). For the rs4253778 polymorphism, when the CC genotype was used as the reference group, the CG and GG genotypes were not associated with dyslipidemia (adjusted OR 0.95, 95% CI 0.63-1.43; adjusted OR0.65, 95% CI 0.24-1.78, respectively). Similar result was observed in the log-additive model(adjusted OR0.89, 95% CI 0.64-1.23). For the rs3856806 polymorphism, when the CC genotype was used as the reference group, the CT and TT genotypes were associated with dyslipidemia (adjusted OR 1.58, 95% CI 1.09-2.27; adjusted OR 2.23, 95% CI 1.28-3.90, respectively). In the log-additive model, rs3856806 also appeared to be associated with dyslipidemia (adjusted OR 1.53; 95% CI 1.19-1.97). For the rs1805192 polymorphism, when the ProPro genotype was used as the reference group, the ProAla and AlaAla genotypes were associated with dyslipidemia (adjusted OR 1.77, 95% CI 1.22-2.56; adjusted OR 2.96, 95% CI 1.70-5.14, respectively). Similar result was observed in the log-additive model (adjusted OR 1.76, 95% CI 1.36-2.27).

**Table 2:** The association analysis between SNPs in PPAR-α/γ and dyslipidemia

**SNP**	**Model**	**Genotype**	**Non- dyslipidemia(N = 628)**	**Dyslipidemia(N = 192)**	**OR (95%CI)**	**P**
rs1800206						
	Codominant	LL	524	98	1.0	<0.001
		LV	99	92	5.75 (3.91-8.46)	
		VV	5	2	2.38 (0.43-13.31)	
	Log additive	-	-	-	4.74 (3.29-6.84)	<0.001
rs4253778						
	Codominant	CC	469	146	1.0	0.670
		CG	133	41	0.95 (0.63-1.43)	
		GG	26	5	0.65 (0.24-1.78)	
	Log additive	-	-	-	0.89 (0.64-1.23)	0.470
rs3856806						
	Codominant	CC	343	75	1.0	0.005
		CT	237	89	1.58 (1.09-2.27)	
		TT	48	28	2.23 (1.28-3.90)	
	Log additive	-	-	-	1.53 (1.19-1.97)	<0.001
rs1805192						
	Codominant	ProPro	380	79	1.0	<0.001
		ProAla	206	82	1.77 (1.22-2.56)	
		AlaAla	42	31	2.96 (1.70-5.14)	
	Log additive	-	-	-	1.76 (1.36-2.27)	<0.001

Adjusted for gender, age, smoke, alcohol, body mass index and waist circumference.

Haplotype association analysis showed the associations between V/G haplotype and dyslipidemia (OR = 5.20; 95%CI = 3.46 ~ 7.82; p < 0.001; Table 3) and between V/G haplotype and TC and TG levels when compared to L/G haplotype (Difference = 0.4, 95% CI = 0.22 ~ 0.59, p < 0.01; Difference = 1.63, 95% CI = 1.31 ~ 1.95, p < 0.01, respectively; Table 2). In addition, when the most common haplotype P-C was treated as the reference group, the P-T, A-C, A-T haplotypes were associated with dyslipidemia (OR = 1.90, 95% CI = 1.33 ~ 2.71, p < 0.001; OR = 2.17, 95% CI = 1.53 ~ 3.09, p < 0.001; OR = 2.06, 95% CI = 1.36 ~ 3.12, p < 0.001, respectively; Table 4). A-T haplotype was associated with TC levels, (Difference = 0.29, 95% CI = 0.09 – 0.48, p < 0.01), and the P-T, A-C, A-T haplotypes were associated with TG levels (Difference = 0.60, 95% CI = 0.32 - 0.88, p < 0.01; Difference = 0.87, 95% CI = 0.58 – 1.17, p < 0.01; Difference = 0.94, 95% CI = 0.57 – 1.30, p < 0.01, respectively) .

**Table 3:** Haplotypes of PPARα rs1800206 and rs4253778 polymorphisms and association with dyslipidemia, TC and TG levels

	**rs1800206**	**rs4253778**	**Frequency**	**OR(95CI%)**	**P-value**
Dyslipidemia	L	G	0.7477	1	-
	L	C	0.1273	1.01 (0. 67 ~ 1.54)	0.94
	V	G	0.1084	5.20 (3.46 ~ 7.82)	<0.001
	V	C	0.0166	2.31 (0.64 ~ 8.32)	0.20
	rs1800206	rs4253778	Frequency	Difference (95CI%)	P-value
Total cholesterol	L	G	0.7487	0	-
	L	C	0.1263	-0.1 (-0.26 ~ 0.05)	0.2
	V	G	0.1074	0.4 (0.22 ~ 0.59)	<0.001
	V	C	0.0176	0.1 (-0.35 ~ 0.55)	0.65
	rs1800206	rs4253778	Frequency	Difference (95CI%)	P-value
Triglyceride	L	G	0.7476	0	-
	L	C	0.1274	0.11 (-0.17 ~ 0.38)	0.45
	V	G	0.1085	1.63 (1.31 ~ 1.95)	<0.001
	V	C	0.0165	0.43 (-0.58 ~ 1.44)	0.4

Adjusted for gender, age, smoking, alcohol consumption, BMI and waist circumference.

**Table 4:** Haplotypes of PPARγ rs1805192 and rs3856806 polymorphisms and association withdyslipidemia, TC and TG levels

	**rs1805192**	**rs3856806**	**Frequency**	**Difference (95CI%)**	**P-value**
Dyslipidemia	Pro	C	0.5393	1	-
	Pro	T	0.196	1.90 (1.33 ~ 2.71)	<0.001
	Ala	C	0.1692	2.17 (1.53 ~ 3.09)	<0.001
	Ala	T	0.0954	2.06 (1.36 ~ 3.12)	<0.001
	rs1805192	rs3856806	Frequency	Difference (95CI%)	P-value
Total cholesterol	Pro	C	0.5395	0	-
	Pro	T	0.1959	0.15 (0.00 ~ 0.30)	0.06
	Ala	C	0.1691	0.09 (-0.07 ~ 0.25)	0.29
	Ala	T	0.0956	0.29 (0.09 ~ 0.48)	<0.001
	rs1805192	rs3856806	Frequency	Difference (95CI%)	P-value
Triglyceride	Pro	C	0.539	0	-
	Pro	T	0.1963	0.6 (0.32 ~ 0.88)	<0.001
	Ala	C	0.1695	0.87 (0.58 ~ 1.17)	<0.001
	Ala	T	0.0951	0.94 (0.57 ~ 1.3)	<0.001

Adjusted for gender, age, smoking, alcohol consumption, BMI and waist circumference.

## Discussion

In this study, we aimed to investigate the association of the four variants at the PPAR α (rs1800206, rs4253778 polymorphisms) and PPAR γ (rs3856806 and rs1805192 polymorphisms) locus with dyslipidemia.

In the single SNP association analysis, we found that rs1800206 polymorphism was associated with dyslipidemia both in the codominant and log-additive models (p < 0.001, Table 2). Rs1800206 polymorphisms has been associated with changes in TG, total cholesterol, LDL- c, HDL- c and APOA1 plasmacon centrations in many populations such as Caucasian, Indian and African-Americans populations [6,7,20,21], in part corroborating our findings. Some previous studies found that the C allele of rs4253778 was associated with increased total and LDL-c, atherosclerosis progression and increased risk for nonfatal myocardial infarction [10,22,23]. Although it seems that the rs4253778 polymorphism has its influence on metabolism disturbances, we did not find associations with dyslipidemia. Interestingly, Mazzotti et al. found divergences on the association results regarding the two polymorphisms between their two studied population (São Paulo City andCuiaba City) [11,12]. For instance, while C allele of rs4253778 was associated with higher HDL-c and lower TG and VLDL levels in the São Paulo population, the same allele was associated with dyslipidemia in the Cuiaba population. Mazzotti et al. [11] considered that the controversy might be explained by the differential population stratification within the two studied samples. These both admixed populations presented different proportions of participants with European and African ancestry, which may account for the differences found between the studies. Moreover, lifestyle and dietary habits differ substantially between these populations. So we thought the divergence on the association result regarding the rs4253778 polymorphisms between our study and other studies might be caused by the same reasons.

Haplotype association analysis showed the associations between V/G haplotype and dyslipidemia (p < 0.001, Table 3) and between V/G haplotype and TC and TG levels (p < 0.01,Table 3) when compared to L/G haplotype (established by rs1800206 and rs4253778). These results confirmed the associations found when individual polymorphisms were analyzed. There is only one study that associated haplotypes of these two polymorphisms with lipid metabolism alterations. Mazzotti et al. [11] found that in the São Paulo population, carriers of the L/C haplotype were at increased risk for dyslipidemia when compared to carriers of the most common L/G haplotype (p = 0.021). In addition, carriers of the V/C haplotype showed a tendency for increased risk for dyslipidemia, but it was not significant (p = 0.055). Unfortunately, the definition for dyslipidemia in the São Paulo population is different from ours. So the associated haplotypes of these two polymorphisms with lipid metabolism alterations should be thoroughly investigated in future studies.

At present, many studies have reported that rs1805192 and rs3856806 polymorphisms were associated with obesity, insulin sensitivity and Type 2 diabetes [16-18]. However, the associations between rs1805192 or rs3856806 polymorphisms and lipid serum levels in the general population were rarely studied. Rs1805192 has been shown to be associated with lower levels of serum total and non-HDL in a Japanese population [24], lower LDL in T2D patients [25], or higher levels of serum HDL-cholesterol in family-based studies [26]. In obesity individuals, this variant was associated with a trend of an increase in TG and significantly associated with the presence of combined hyperlipidaemia [27]. In a group of 57 extremely obese men, this variant was related with an increase in TG. In CAD patients, Zhou et al. [28] found that rs3856806 polymorphism was associated with a higher HDL cholesterol level and a lower blood glucose level. Yilmaz-Aydogan [29] found that subjects with the CT heterozygote genotype of the rs3856806 polymorphism showed a higher serum triglyceride and VLDL-C concentrations than those with the CC homozgotes in the CHD patients with diabetes. Our study found that rs1805192 and rs3856806 polymorphisms were associated with dyslipidemia both in the codominant and log-additive models (p <0.01; Table 2), suggesting an independent association between the two polymorphisms and dyslipidemia.

Haplotype analysis regarding rs1805192 and rs3856806 polymorphisms has also shown the associations with dyslipidemia, TC and TG levels. When compared to carriers of the most common Pro/C haplotype, the Pro-T, Ala-C, Ala-T haplotypes were associated with dyslipidemia (p < 0.001, Table 4). In addition, Ala-T haplotype was associated with higher TC levels, (p < 0.01), and the Pro-T, Ala-C, Ala-T haplotypes were associated with higher TG levels (p < 0.01). So far, the haplotype analysis regarding rs1805192 and rs3856806 polymorphisms for dyslipidemia has not been reported yet.

Our study found that PPAR α and PPAR γ haplotypes were associated with dyslipidemia. PPAR α controls the expression of a wide range of hepatic genes encoding for proteins involved in fatty acid catabolism and lipoprotein metabolism. Its activation leads to changes in the transcription of multiple genes that regulate lipid and lipoprotein metabolism including LPL, APOC3, APOA1 and APOA5. Vohl [9] and Sapone [30] et al. found that rs1800206 had functional consequences on receptor activity. PPAR γ plays an indispensible role in the regulation of adipocyte differentiation, lipid storage, glucose metabolism and the transcriptional regulation of a number of genes involved in these metabolic processes. The key target genes of PPAR γ include the fat-specific ap2 gene, LPL, fatty acid transport, fatty acidbinding protein, ABC-A1 and so on [6]. Deeb [31] found that the Ala allele of rs1805192 showed decreased binding affinity to the cognate promoter element and reduced ability to transactivate responsive promoters. rs3856806 was found to modulate the associations observed with the codon 12 substitution [15]. We thought rs1800206, rs1805192 and rs3856806 polymorphisms may influence the receptor activity, the ability to transactivate responsive promoters and so on to regulate the key target genes of PPAR α/γ which could influnce the lipid metabolism. But the specific biological mechanism needs to be further studied.

One limitation of this study is that these findings may not be generalizable to other populations. Large ethnically matched studies would be necessary to know if such reports are found in non-Chinese Han subjects. Second, the information on some lifestyle and dietary habits which might interact with PPAR α/γ genotypes or act as potential confounding factors was not available in our study.

In conclusion, our study has tested the association between four common polymorphisms within PPAR α/γ gene and dyslipidemia based on single-locus and haplotype analyses. These results may help to evaluate their polymorphisms and haplotypes as being characterized as genetic risk factors for dyslipidemia. Independent replications in large sample sizes are needed to confirm the role of the polymorphisms and haplotypes found in this study and the risk factors such as the lifestyle and dietary habits should be thoroughly investigated in future studies.

## Materials and methods

### Studied population

Participants were recruited from the Prevention of MetS and Multi-metabolic Disorders in Jiangsu Province of China Study [32], which was initiated from April of 1999 to June of 2004. Totaled 4,582 subjects for whom five year follow-up data were obtained between March of 2006 and October of 2007. A total of 4,083 participants (89.11%) received follow-up examination (the patients who attended the follow-up examinations were similar to those lost from the follow-up cohort in terms of age, sex, smoking status, alcohol intake, family disease history and metabolic variables; p >0.05). After excluding subjects who had experienced stroke or exhibited cardiovascular disease (n = 36, 11 of whom died), type 2 diabetes (n = 289, 31 of whom died), missing data (n = 133) or body mass index (BMI) < 18.5 kg/m^2^ (n = 27, 2 of whom died), a total of 820 unrelated individual subjects (270 males, 550 females) were was selected from the remaining 3,731 cases by using simple random sampling and no individual was related. Subjects who were selected were similar to those who were not selected in terms of age, sex, smoking, alcohol, family disease history and metabolic variable. Blood samples of the 820 subjects were collected as a baseline and subjected to genotype analyses. Lipids measured at follow-up were used to examine the association with PPAR γ polymorphisms in the study. All the participants signed the informed consent form. The study was approved by the ethic committee of Soochow University.

### Body measurements and laboratory methods

Participants in baseline and follow-up study surveys both filled in a standard questionnaire and physical measurements and were collected blood samples in the morning after at least 8 hours of fasting (used to detect and establish cell library). The methods of investigation at the follow up were same as those at baseline. Cigarette smokers were those who reported smoking cigarettes at least once a day for 1 year or more. Total alcohol intake was expressed as the sum of milliliters of alcohol per week from wine, beer and spirits. The anthropometric measurements included body weight and waist circumferences (WC). BMI was calculated according to the Quetelet equation. Plasma glucose was measured using the oxidase enzymatic method. Serum total cholesterol (TC), HDL-c, and TG levels were measured by enzymatically. LDL-c concen-trations were calculated by using the Friedewald formula. The measurements were conducted using an automatic biochemistry analyzer (Hitachi Inc, Tokyo Japan). All analyses were performed by the same lab. The methods of investigation at the follow up were same as those at baseline.

### Genotyping

Genomic DNA from participants was extracted from EDTA-treated whole blood, using the DNA Blood Mini Kit (Qiagen, Hilden, Germany) according to the manufacturer’s instructions. The qualities of isolated genomic DNAs were checked using agarose gel electrophoresis and the quantities determined using spectrophotometry. Two approaches were employed to separate wildtype and mutated alleles for the four SNPs. rs4253778 was detected by plymerase chain reaction restriction fragment length polymorphisms (PCR-RFLP), and the left three SNPs were detected by Taqman fluorescence probe. Restriction enzyme was used to identify and cut specific sequence, then PCR was performed with the following primers: forward 5′- ACA ATC ACT CCT TAA ATA TGG TGG-3′ and reverse 5′- AAG TAG GGA CAG ACA GGA CCA GTA -3′. A 25 μl reaction mixture was amplified by PCR, including DNA 20 ng, 0.05 μl Ex Taq DNA polymerase, 1 μl 10 × buffer, 0.8 μl dTNP, 0.1 μl Forward primers, 0.1 μl reverse primers. PCR conditions were as follows: initial denaturation for 3 min and 95°C, denaturation for 10 s and 95°C, annealing for 30 s and 63°C, extension for 30 s and 72°C, 40 cycles. ABI Prism7000 software and allelic discrimination procedure was used for genotyping of fore-mentioned four SNPs. A 25 μl reaction mixture including 1.25ul SNP Genotyping Assays(20×), 12.5 μl Genotying Master Mix(2×), 20 ng DNA, and the conditions were as follows: initial denaturation for 10 min and 95°C, denaturation for 15 s and 92°C, annealing and extension for 90 s and 60°C, 50 cycles. Table 5 provides detailed information on the selected SNPs, including their features, allelic variants, and the minor allele frequencies.

**Table 5:** Description for 4 SNPs of PPAR-α/γ

**SNP ID**	**Chromosome**	**Position**	**Exon/Intron**	**Substitution**	**MAF**^**a**^
PPAR α					
rs4253778	22	26021203	Intron_7	G > C	0.22
rs1800206	22	26004843	Exon_5	L > V	0.13
PPAR γ					
rs1805192	3	12361238	Exon_B	Pro > Ala	0.26
rs3856806	3	12415557	Exon-6	C > T	0.15

^a^MAF in the total group of this study.

MAF, minor allele frequency; SNP, single nucleotide polymorphism.

### Definitions

The criteria for lipid and lipoprotein levels were according to the National Cholesterol Education Program [33]. Participants were diagnosed with dyslipidemia if they had one or more of the following criteria: a plasma concentration of TC of ≥6.24 mmol/L, plasma concentration of TG ≥ 2.26 mmol/L; plasma concentration of HDL-c of <1.04 mmol/L for men or <1.30 mmol/L for women; and a plasma concentration of LDL-c ≥4.14 mmol/L.

### Statistical analyses

The mean and standard deviation (SD) for continuous variables normally distributed and percentage for categorical variable were calculated. Median and inter-quartile range were calculated for continuous variables that were not normally distributed. Continuous variables were tested using the t-test and no-parametric test. Frequencies of categorical variables were tested using the chi-square test. Allele and genotype frequencies were calculated for each polymorphism and the χ2test was used to investigate deviation from Hardy –Weinberg equilibrium. The logistic regression model was used to examine the association between PPARα/γ polymorphisms and dyslipidemia. Odds-ratio (OR) and 95% confidence interval (95% CI) were also calculated. Individual polymorphism data analyses were performed using SPSS 16.0. Estimation of linkage disequilibrium between polymorphisms was performed using SHEsis (http://analysis2.bio-x.cn/SHEsisMain.htm) software. Haplotype association analysis was performed using SNPStats web tool.

## Abbreviations

CVD: Cardiovascular disease; LDL-c: LDL-cholesterol; TG: Triglycerides; HDL-c: HDL-cholesterol; PPAR: Peroxisome proliferator-activated receptor; BMI: Body mass index; WC: Waist circumferences; TC: Total cholesterol; PCR-RFLP: Plymerase chain reaction restriction fragment length polymorphisms; SD: Standard deviation; OR: Odds-ratio; CI: Confidence interval.

## Competing interests

The authors declare no conflicts of interests.

## Authors’ contributions

ZRG, XSH and ZYZ designed research; ZYZ and MW coordinated and completed the trial and collected all the data; SJG performed the laboratory analysis, compiled data and performed the statistical analysis; ZRG and SJG wrote the final draft and had primary responsibility for the final conduct. All authors read and approved the final manuscript.
